# Advanced Deep Learning Approach to Automatically Segment Malignant Tumors and Ablation Zone in the Liver With Contrast-Enhanced CT

**DOI:** 10.3389/fonc.2021.669437

**Published:** 2021-07-15

**Authors:** Kan He, Xiaoming Liu, Rahil Shahzad, Robert Reimer, Frank Thiele, Julius Niehoff, Christian Wybranski, Alexander C. Bunck, Huimao Zhang, Michael Perkuhn

**Affiliations:** ^1^ Department of Radiology, The First Hospital of Jilin University, Changchun, China; ^2^ College of Electronic Science and Engineering, Jilin University, Changchun, China; ^3^ Innovative Technologies, Philips Healthcare, Aachen, Germany; ^4^ Institute for Diagnostic and Interventional Radiology, Faculty of Medicine and University Hospital Cologne, University of Cologne, Cologne, Germany

**Keywords:** liver cancer, ablation zone, segmentation, computed tomography, U-Net

## Abstract

**Objective:**

Liver cancer is one of the most commonly diagnosed cancer, and energy-based tumor ablation is a widely accepted treatment. Automatic and robust segmentation of liver tumors and ablation zones would facilitate the evaluation of treatment success. The purpose of this study was to develop and evaluate an automatic deep learning based method for (1) segmentation of liver and liver tumors in both arterial and portal venous phase for pre-treatment CT, and (2) segmentation of liver and ablation zones in both arterial and portal venous phase for after ablation treatment.

**Materials and Methods:**

252 CT images from 63 patients undergoing liver tumor ablation at a large University Hospital were retrospectively included; each patient had pre-treatment and post-treatment multi-phase CT images. 3D voxel-wise manual segmentation of the liver, tumors and ablation region by the radiologist provided reference standard. Deep learning models for liver and lesion segmentation were initially trained on the public Liver Tumor Segmentation Challenge (LiTS) dataset to obtain base models. Then, transfer learning was applied to adapt the base models on the clinical training-set, to obtain tumor and ablation segmentation models both for arterial and portal venous phase images. For modeling, 2D residual-attention Unet (RA-Unet) was employed for liver segmentation and a multi-scale patch-based 3D RA-Unet for tumor and ablation segmentation.

**Results:**

On the independent test-set, the proposed method achieved a dice similarity coefficient (DSC) of 0.96 and 0.95 for liver segmentation on arterial and portal venous phase, respectively. For liver tumors, the model on arterial phase achieved detection sensitivity of 71%, DSC of 0.64, and on portal venous phase sensitivity of 82%, DSC of 0.73. For liver tumors >0.5cm^3^ performance improved to sensitivity 79%, DSC 0.65 on arterial phase and, sensitivity 86%, DSC 0.72 on portal venous phase. For ablation zone, the model on arterial phase achieved detection sensitivity of 90%, DSC of 0.83, and on portal venous phase sensitivity of 90%, DSC of 0.89.

**Conclusion:**

The proposed deep learning approach can provide automated segmentation of liver tumors and ablation zones on multi-phase (arterial and portal venous) and multi-time-point (before and after treatment) CT enabling quantitative evaluation of treatment success.

## Introduction

Liver cancer is one of the most commonly diagnosed cancer globally in 2018, with about 841.000 new cases and 782.000 deaths annually (1). Hepatocellular carcinoma (HCC) is the most frequent primary liver cancer and the third leading cause of cancer death (2). Liver metastases, which are secondary liver cancer, may occur in the early stages of gastrointestinal malignancy because of hematogenous spread through the portal venous system (3, 4). The morbidity and mortality of these populations with liver cancer increase significantly.

Given the complexity of liver cancer and many potentially useful treatments, the most appropriate treatment option should be selected for each patient at each tumor stage. Energy-based tumor ablation has become a widely accepted treatment option for patients with early-stage liver cancer in recent years. Several energy-based ablation technologies are currently available, including radiofrequency ablation (RFA), microwave ablation (MWA), laser ablation, and cryoablation. Before and after energy-based ablation, cross-sectional imaging is necessary to plan the ablation and to assess treatment response. The most used modality for follow-up is multi-phase contrast-enhanced computed tomography (CT) due to its broad availability, robustness, and high reproducibility (5). RFA and MWA aim to achieve irreversible cellular injury and cellular death, leading to eradicating the target tumor (6). During the ablation treatment, the segmentation is a crucial first step that provides a series of quantitative measurements, including volume, shape, localization, and the proportion for liver or lesion.

However, precise segmentation of liver tumors or the ablation zone is still challenging in planning ablation before the procedure and assessing treatment response after the procedure. Some studies have assessed the ablation treatment by subjective estimations and manual segmentation (7–9). This kind of method is time-consuming and suffers from low consistency and reproducibility. It heavily depends upon prior human knowledge, and it requires people with high-level of skills to accomplish such tasks. For more effective and consistent target lesion identification and treatment evaluation, automatic delineation of the liver cancer, ablation zone, and liver organ is necessary, which holds great promise for enhancing radiology workflow. Nevertheless, lesion and organ segmentation are still challenging. Up until now, some computer-aided diagnosis (CAD) strategies are applied to segment the regions of interest (ROIs) automatically. Initially, some classical image processing methods such as region growing (10), graph-cut (11), and level-set (12) were applied to liver tumor segmentation.

Lately, deep learning (DL) has shown promising performances in many fields. DL techniques have several advantages over conventional frameworks. Feature extraction, selection, and supervised classification can be realized within the same deep architecture. With such a design, the performance can be tuned more efficiently in a systematic way (13). Among many DL architectures, convolutional neural networks (CNN) especially U-Net (14) has been regarded as a powerful CNN to deal with liver tumors’ segmentation tasks (15, 16). Some strategies, such as residual modules (17), dense connection (18) and adversarial training (19) are integrated into U-Net to improve the segmentation accuracy of liver tumors. However, segmenting the tumor is still challenging, mainly due to the uncertain number and location of lesions. Some researchers (20–22) have reported that combined liver and tumor segmentation will improve the performances.

In this paper, we present an automatic method based on DL to realize accurate segmentation of the liver, tumors and ablation zones in multi-phase CT images of liver cancer patients and thus make a quantitative efficacy evaluation for RFA/MWA. In our study, we used the public Liver Tumor Segmentation Challenge (LiTS) (23) dataset and a clinical dataset from our hospital on patients undergoing microwave ablation to evaluate the proposed method.

## Materials and Methods

### Data Sources and Patient Demographics

The employed datasets consist of two parts. Firstly, we used the LiTS dataset and segmentations provided by various clinical sites worldwide. LiTS includes 130 CT images of portal venous phase in patients with HCC or secondary liver tumors.

Secondly, we retrospectively collected multi-phase CT images (at initial and follow-up imaging) between 2010 and 2019 in patients who underwent RFA or MWA at our institution. The local Institutional Review Board approved this retrospective, single-center study and waived the requirement for written informed consent for the patient cohort. 104 patients were identified, applying the following eligibility criteria: (1) patients (≥ 18 years) who were referred to our radiology department for liver RFA/MWA, (2) pathology proven HCC or liver metastases, (3) complete multi-phase CT images, arterial phase and portal venous phase, and (4) CT images of patients with both pre- and post-procedure. Targeted lesions that were not visualized well due to artefacts or patients with follow-up interval < 1 week were excluded from this study. In total, 63 patients with 252 CT images were identified. CT contrast images were acquired on iCT 256, Brilliance 64, IQon, Somatom Definition FLASH scanners (Philips and Siemens) with 2 mm slice thickness. Contrast enhanced CT of the liver can obtain dynamic post-contrast images in multiple phases. The arterial phase begins approximately 15-25 seconds following intravenous contrast administration, portal venous phase about 50-70seconds.The institutional CT protocol for tumor evaluation before and after energy-based ablation comprises an arterial and a portal venous phase of the liver. After administration of 100 ml non-ionic, iodinated contrast media bolus (Accupaque 350 mg/ml, GE Healthcare) followed by a 30 ml saline chaser with an automated injection system at a flow rate of 3.5 ml/s (MEDRAD^®^ Stellant^®^, Bayer Vital AG), bolus-tracking technique (threshold of 150 Hounsfield Units (HU) in the abdominal aorta) is used, and image acquisition starts with a delay of 15 and 50 s, respectively. The identified patients were randomly divided into a training set consisting of 48 patients and a test set of 15 patients, ensuring no overlap of data between the 2 sets. The training data was used for DL training and the test set used for independent testing of the DL models. Demographics and other characteristics of the local clinical cohort are summarized in **Table 1**.

**Table 1:** Characteristics of 63 patients in local clinical dataset.

Clinical characteristics	Values
Age (years)	
Mean ± SD	64.3 ± 10.9
Range	32-83
Gender	
Male	47
Female	16
Cirrhosis	28
Etiology	
HBV	5
HCV	11
NASH	5
Alcoholic liver disease	15
Others	27
Treatment for patients	
MWA	53
RFA	10
Tumor number per patient	
n=1	46
n=2	11
n=3	6
Pathology of tumor	
HCC	41
Metastases	45
Tumor size(cm)	
Mean ± SD	2.08 ± 0.92
Range	0.67-4.92
Tumor volume(cm^3^)	
Mean ± SD	10.43 ± 18.52
Range	0.06-82.61
Ablation zone size(cm)	
Mean ± SD	5.73 ± 1.22
Range	2.62-8.84
Ablation zone volume(cm^3^)	
Mean ± SD	56.66 ± 36.20
Range	6.76-200.78

### Reference Standard

Segmentation of liver masks, tumors and ablation zones in the arterial phase and portal venous phase from 252 CT images used as reference standard were created manually using IntelliSpace Discovery (ISD, v. 3.0.6, Philips Healthcare, Best, The Netherlands) by two radiologists (H.K and R.R), with 9 and 4 years of experience in liver imaging, respectively. To establish the reference standard, both readers reviewed all images and segmentations in LiTS data together and then performed segmentations on the local clinical dataset in a 3D voxel-wise manner on ISD using consensus reading.

### Data Pre-Processing

For CT images, the relative densities are measured by Hounsfield units (HU). To remove the influence of other organs or tissues around the liver, we firstly performed clipping in the range -100HU to 200HU. Then we resampled all the images to an isotropic resolution of 1.0 × 1.0 × 1.0 mm^3^. Data augmentation such as random flipping and rotations were applied during training to increase data samples on the fly.

### Residual Attention U-Net

In this study, we used a residual attention U-net (RA-Unet) as the architecture to segment the CT images. This network (24) is inspired by U-net (14) and residual attention mechanism (25). Traditional U-net consists of an encoder and a decoder, encoder extracts hierarchical features of the input image while decoder reconstructs the features in a coarse-to-fine manner. Skip-connections combines different-scale features from these two parts. Residual blocks are introduced to replace the initial convolutional layers to increase the depth of the network and avoid gradient vanishing. Furthermore, attention blocks are added to skip-connections in order to pay more attention to the important regions in the coarse features from the encoder. There are two paths in each attention block, the first path performs feature processing, and the second path collects and combines the global information from the whole image also by an encode-decoder module. Improvement of RA-Unet’s performance compared to conventional U-net has been previously demonstrated (26).

2D convolutions are used in 2D RA-Unet and 3D convolutions in 3D RA-Unet. In each architecture, 9 residual blocks with kernel of size 3×3 or 3×3×3 and 4 attention blocks are present. The residual blocks have a skip connection and 3 stacks of batch normalization, activation function and convolution layers each. Identity mapping is performed by the skip connection, the output of the identity mapping is added to the output of the stacked layers (27). The activation function used in the residual blocks as well as the rest of the network is ReLu except for the final layer of the network where a sigmoid function is used to produce a binary segmentation. Loss function used was dice loss and is provided as:

$$L_{dice}=1-\frac{2\;\Sigma s_{true}*s_{pred}}{\Sigma s_{true}^{2}+\Sigma s_{pred}^{2}}$$

where *S_true_* and *S_pred_* are the ground truth and predicted binary segmentations respectively.

The training of the network was initiated with random values, Adam optimizer was used with a learning rate of 0.0001. The number of training epochs was set to 1500. Early stopping with 500 epochs was used to avoid over-training. Adaptive learning rate was utilized by reducing the learning rate by a factor of 10% if the network plateaus after 20 epochs.

**Figure 1:**
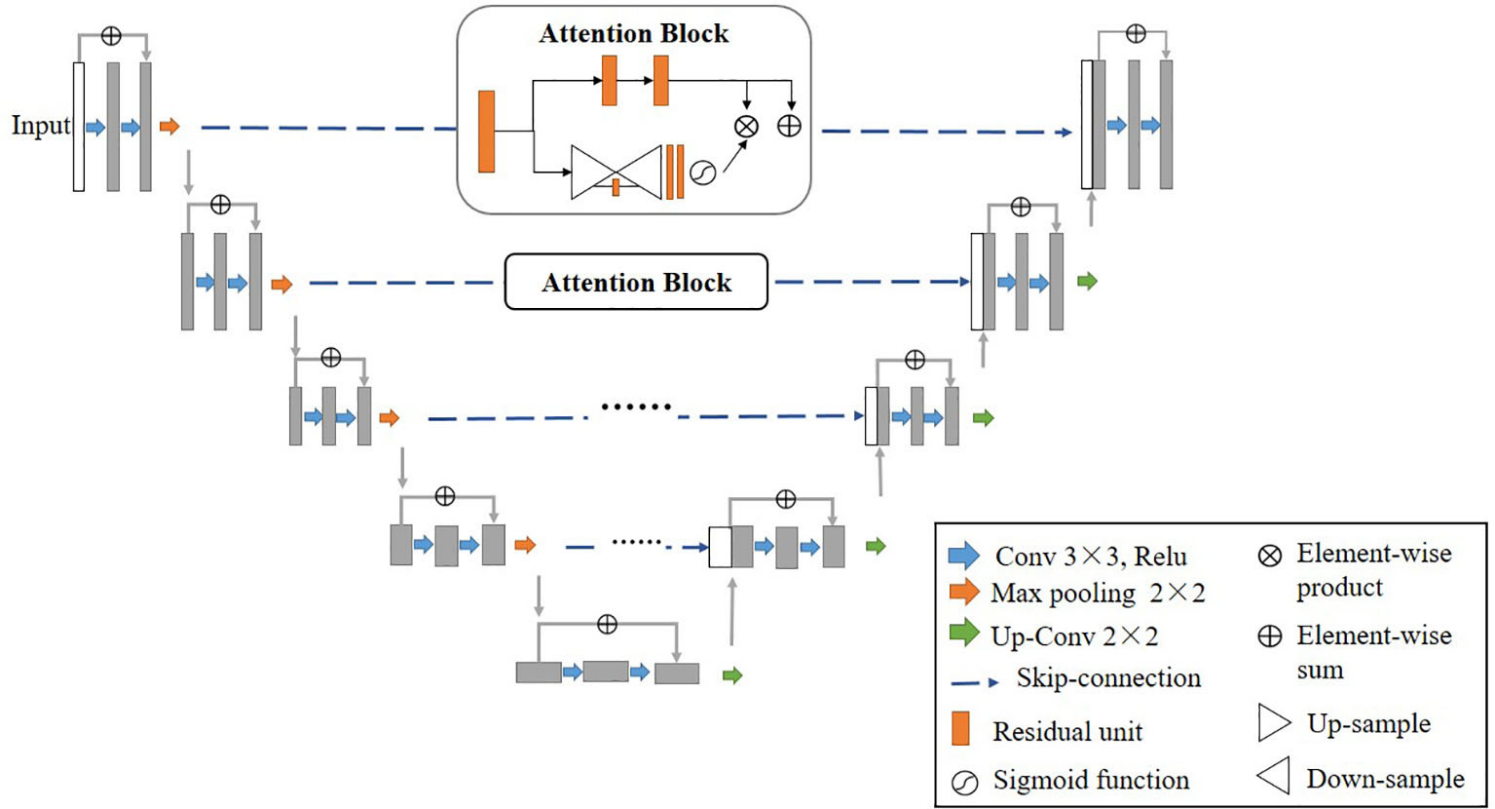
Architecture of residual attention U-net (RA-Unet).

**Figure 1** represents the architecture of the RA-Unet and **Table 2** shows the network parameters for the 2D RA-Unet. The network has a total of 2.9 million parameters.

**Table 2:** Architecture of the 2D RA-Unet used for liver segmentation.

Encoder	Output size	Decoder	Output size
Input	512×512×1	Attention block 1	64×64×256
Residual block 1	512×512×32	Residual block 6	64×64×256
Pooling	256×256×32	Up convolution	128×128×256
Residual block 2	256×256×64	Attention block 2	128×128×128
Pooling	128×128×64	Residual block 7	128×128×128
Residual block 3	128×128×128	Up convolution	256×256×128
Pooling	64×64×128	Attention block 3	256×256×64
Residual block 4	64×64×256	Residual block 8	256×256×64
Pooling	32×32×256	Up convolution	512×512×64
Residual block 5	32×32×512	Attention block 4	512×512×1
Up convolution	64×64×512	Residual block 9	512×512×1

### Liver Segmentation

2D RA-Unet was used to segment the liver. 2D RA-Unet was initially trained on the portal venous phase images from the LiTS dataset, to obtain a base model. The network was subsequently trained on the local clinical dataset, which included CT images in the arterial phase and portal venous phase from pre- and post-ablation treatment. Transfer-learning approach (28) was used to help facilitate model training on the local clinical dataset. By using a transfer learning approach, the layer weights from the base model were fine-tuned on the local clinical dataset. Unlike using random values that were used as initialization to train the base model. By using transfer learning the model learned to segment both CT phases. During training of liver segmentation, batch size was set to 10 slices.

### Tumor and Ablation Zone Segmentation

Two dedicated 3D RA-Unet models were trained for segmentation of tumors and ablation zone, respectively. In order to compensate for the varying numbers and sizes of lesions we built an ensemble model using multi-scale inputs to the 3D RA-Unet to improve segmentation effectiveness. The multi-scale inputs were 3D patches of size 20×30×30 and 40×60×60, extracted from within the liver. The liver mask was obtained using the liver segmentation model. Similar to the liver segmentation model, the multi-scale tumor model was first pre-trained on the portal venous phase images from the LiTS dataset and then retrained on the arterial and portal venous phases of the clinical dataset using transfer-learning, which enabled the model to efficiently learn how to segment the tumor lesions on both CT phases. During training, batch size was set to 72 3D patches, class balance was ensured by choosing equal number of foreground and background patches. All experiments were performed on a Linux workstation with two Tesla V100 GPU’s (NVIDIA, Santa Clara, USA). The training and testing for models of liver segmentation, tumor segmentation and ablation zone segmentation were developed using Keras (2.2.4) with TensorFlow (1.15.2) backend.

### Inference

In the test phase, we first performed the same preprocessing as the training phase, including clipping and resampling. Then, the liver is segmented using the 2D RA-Unet model. This is followed by extracting a bounding box of size 200×300×300 around the liver mask. Next, 2 sets of 3D patches of size 20×30×30 and 40×60×60 with 10% overlap are extracted from the bounding box. The 3D patches are passed to the respective 3D RA-Unet models for tumor or ablation zone prediction. Finally, the resulting output segmentations are stitched together according to the original order. The overlapping segmented voxels from each 3D patch were retained and merged into the final segmentation outcome for the tumor or ablation zone.

### Statistical Analysis

To evaluate the performance of the DL models as compared to the reference standard, spatial overlap is measured using dice similarity coefficient (DSC):

$$DSC=\frac{2\cdot \left|RS\cap PS\right|}{\left|RS\right|\cup \left|PS\right|}$$

where RS is the reference standard and PS is the predicted segmentation.

Additionally, to evaluate the detection accuracy of the liver tumors and the ablation zone the following measures are used:

$$Sensitivity=\frac{TP}{TP+FN}$$

$$Precision=\frac{TP}{TP+FP}$$

$$F1\;Score=\frac{2*Sensitivity*Precision}{Sensitivity+Precision}$$

where TP are the true positives, FN are the false negatives and FP are the false positives.

For quantitative volumetric measurements, Pearson’s correlation coefficient (r) was calculated and Bland–Altman analysis was performed.

Sub-analysis was performed for tumors of volume ≥ 0.5 cm^3^, which corresponds to spherical tumors with diameter > 1cm.

## Results

### Clinical Characteristics

The LiTS dataset included 130 CT images (283 lesions) of portal venous phase in patients with HCC or secondary liver tumors. The local clinical dataset included 63 patients with 252 CT images of the arterial phase and portal venous phase. The median age of the patients was 64.3 years (range, 32–83) and males were dominant (74.6%) (**Table 1**). Forty-one treated tumors (47.7%) were HCC, and forty-five tumors (52.3%) metastases. Mean tumor size and tumor volume, based on manual segmentation, were 2.08 **±** 0.92 cm and 10.43 **±** 18.52 cm^3^, respectively. Mean ablation zone size and volume, based on manual segmentation, were 5.73 **±** 1.22 cm and 56.66 **±** 36.20 cm^3^, respectively. The 63 patients were randomly divided into a training set consisting of 48 patients and a test set consisting of 15 patients. The training set had 59 tumors with a mean volume of 9.74 **±** 19.65 cm^3^ and 53 ablation zones with a mean volume of 49.63 **±** 31.11 cm^3^. Whereas the test set had 27 tumors with a mean volume of 11.98 **±** 16.05 cm^3^ and 18 ablation zones with a mean volume of 71.91 **±** 42.35 cm^3^.

### Results for Liver Segmentation

**Figure 2:**
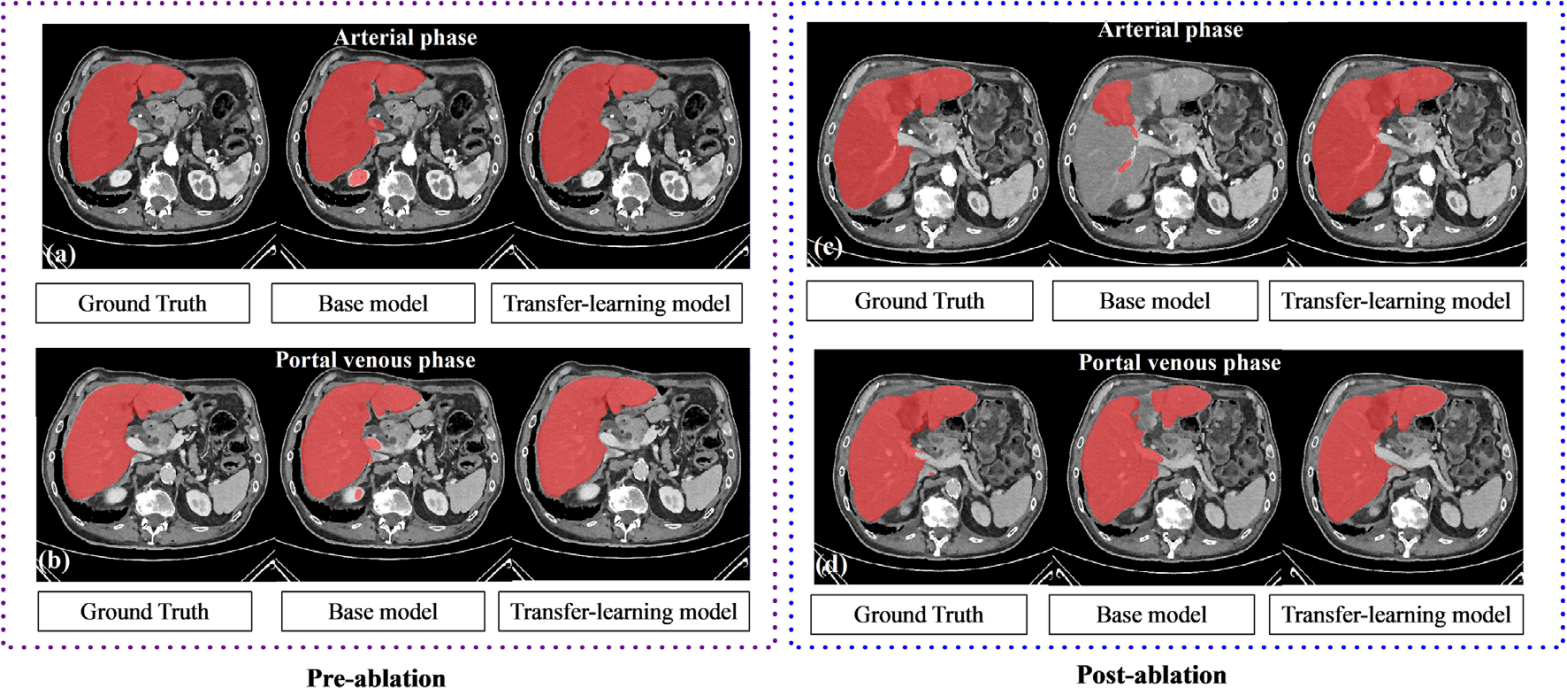
The performance of liver segmentation in base model and transfer-learning model. Transfer learning model performs better on the local clinical data set, especially in the arterial phase.

Results of the liver segmentation model are depicted in **Table 3**. It was observed that the transfer-learning model performs better than the base model. Segmentation improvements were pronounced on the arterial phase, where the DSC increases from 0.89 ± 0.09 to 0.95 ± 0.01 for the pre-ablation group, and from 0.92 ± 0.14 to 0.96 ± 0.01 for the post-ablation group. **Figure 2** shows an example of the liver segmentation performance using the base model as well as the transfer-learning model.

**Table 3:** Results of liver segmentation model in clinical dataset.

	Pre-ablation		Post-ablation	
	Arterial phase	Portal venous phase	Arterial phase	Portal venous phase
	median dice ± std	median dice ± std	median dice ± std	median dice ± std
Base model	0.89 ± 0.09	0.94 ± 0.05	0.92 ± 0.14	0.93 ± 0.02
Transfer-learning model	0.95 ± 0.01	0.95 ± 0.01	0.96 ± 0.01	0.95 ± 0.01

### Results for Tumor Segmentation

In the pre-ablation group, the multi-scale tumor segmentation ensemble model had a sensitivity of 71% on the arterial phase and 82% on the portal venous phase. The ensemble model’s median DSC on the venous phase was 0.73 and was higher than that on the arterial phase which was 0.64, see **Table 4** for detailed results. For lesions > 0.5cm^3^ we observed a dramatic drop in FP’s/image and enhanced precision and F1 score both for arterial phase (n=15, precision from 46% to 71%, F1 score from 0.56 to 0.75) and portal venous phase (n=24, precision from 44% to 74%, F1 score from 0.57 to 0.79). Comparing volumetric assessment between the automated and manual segmentations, Pearson correlation coefficients (r) were: 0.85 for tumor segmentation in arterial phase, 0.70 in portal venous phase.

**Table 4:** Results of tumor segmentation model.

	Median dice	r	Sensitivity	Precision	F1 score	FP’s/image
Arterial phase	0.64	0.85	71%	46%	0.56	1.33
Arterial phase	0.65	0.84	79%	71%	0.75	0.4
(>0.5cm^3^)						
Portal venous phase	0.73	0.70	82%	44%	0.57	2.33
Portal venous phase	0.72	0.68	86%	74%	0.79	0.6
(>0.5cm^3^)						

### Results for Ablation Zone Segmentation

**Figure 3:**
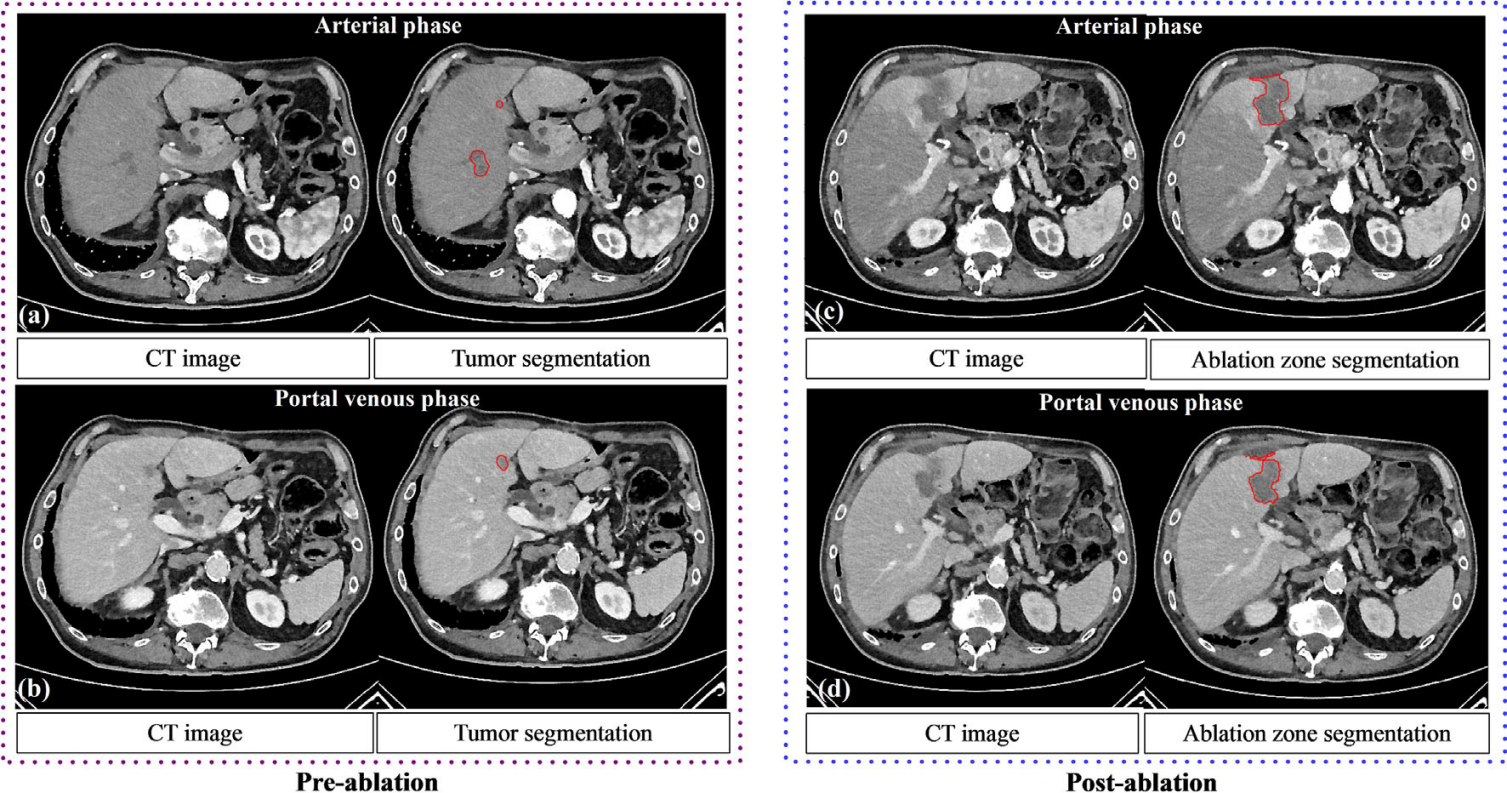
The performance of tumor segmentation and ablation zone segmentation. Pre **(A, B)** and post **(C, D)** ablation contrast CT of a 65-year-old male patient with liver metastases. Tumor and ablation segmentations using the deep learning model are shown.

**Figure 4:**
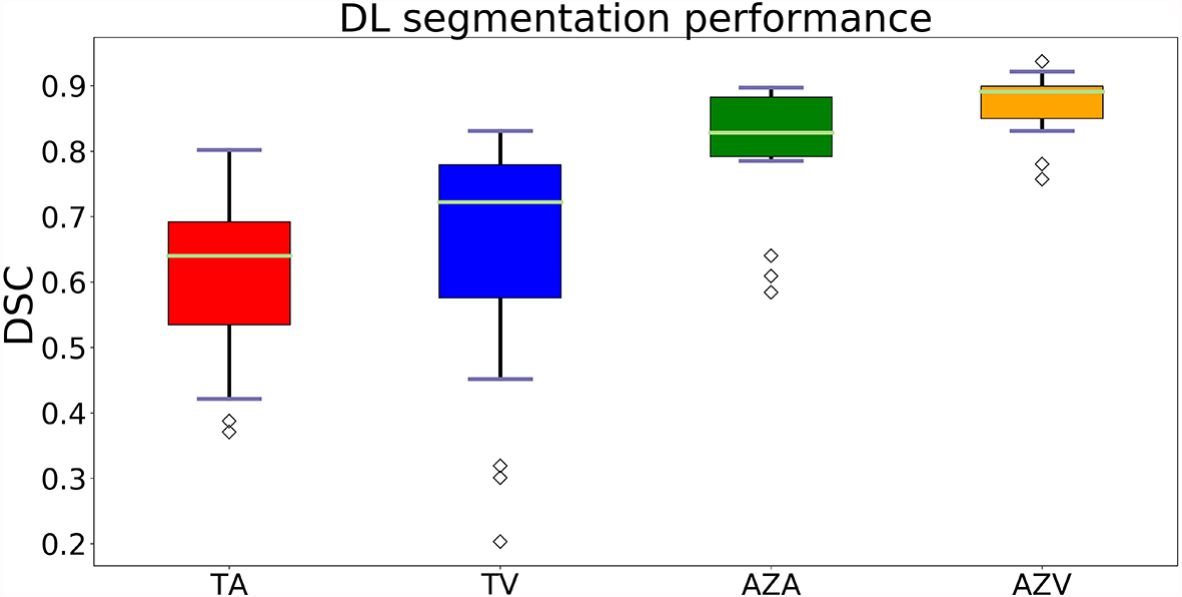
Box-plots of DSC’s showing the accuracy of the segmentations. Where TA, tumors on arterial phase; TV, tumors on the portal venous phase; AZA, ablation zones on arterial phase and AZV, ablation zones on portal venous phase.

**Figure 5:**
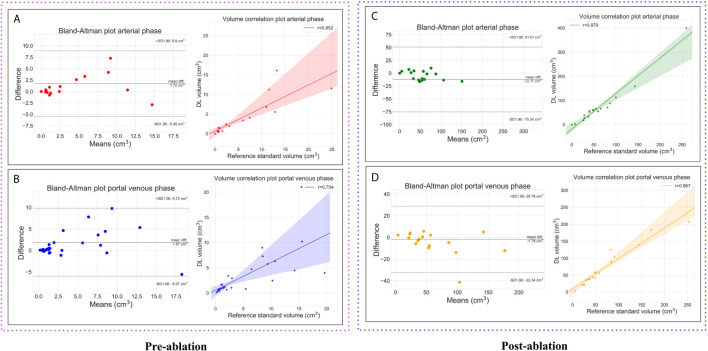
Plots showing the volumetric assessment between the reference standard and the automatic deep learning based segmentations. Lesions on pre-ablation arterial phase **(A)**, portal venous phase **(B)** and, ablation zones on the post-ablation arterial phase **(C)**, portal venous phase **(D)**.

In the post-ablation group, the multi-scale ablation zone segmentation ensemble model had a sensitivity of 90%, both on the arterial and portal venous phase. Median dice of 0.83 and 0.89 and a F1 score of 0.74 and 0.73 were noted for arterial and portal venous phase, respectively. Pearson correlation coefficients (r) were: 0.98 for ablation zone segmentation in arterial phase, and 0.97 in portal venous phase. More detailed results are presented in **Table 5**. **Figure 3** is an example to show the tumor and ablation zone segmentation visually. **Figure 4** shows DSC box-plots for lesions and ablation zones. Bland-Altman analysis plots and correlation plots are shown in **Figure 5**.

**Table 5:** Results of ablation zone segmentation model.

	Median dice	r	Sensitivity	Precision	F1 score	FP’s/image
Arterial phase	0.83	0.98	90%	58%	0.74	1.0
Portal venous phase	0.8	0.97	90%	61%	0.73	0.8

## Discussion

In this study, we developed and trained a DL method for segmenting the liver, liver tumors and ablation zones on multi-phase CT images. The RA U-net based DL models were initially trained on the public MICCAI 2017 LiTS dataset and then applied to the local clinical dataset using transfer learning. The models were evaluated on an independent test-set including both pre-ablation and post-ablation CT images.

In our study, we obtained a median DSC of 0.95 for the pre-ablation group, and 0.96 for the post-ablation group on liver segmentation; 0.64 for arterial phase, and 0.72 for portal venous phase on liver tumor segmentation. The method provided high segmentation performance on a clinical routine dataset. Furthermore, to improve the performance of tumor and ablation zone segmentation, a multi-scale ensembling strategy was introduced. The established models trained on two patch sizes could better capture the contextual information and reduce the number of FP’s/images, which effectively enhances the ability to segment lesions of different sizes. Additionally, we found that the per-lesion sensitivity of automated tumor detection is significantly higher for tumors with volume greater than 0.5 cm^3^, with sensitivities improving from 71% and 82% to 79% and 86% on arterial phase and portal venous phase, respectively. For these tumors, we also observed a dramatic increase in segmentation accuracy, precision, F1 score, and a drop in FP cases.

The extraction of liver and tumors from CT is critical before and after RFA/MWA in choosing an optimal approach for the treatment and evaluating for treatment success. The incorporation of the automatic segmentation method into the clinical ablation workflow could improve the performance of image analysis by decreasing inter-observer variation and providing the quantitative support for the minimal ablative margin (MAM) assessment. However, due to the heterogeneous and diffusive liver and tumor shapes, segmenting them from the CT images is quite challenging. Numerous efforts have been taken to tackle the segmentation task on liver/tumors. Christ et al. (29) reported an accuracy of 65% with a DSC of 0.69 for HCC segmentation using two CNN architectures. Using a fully automatic 2-stage cascaded method based on the LiTS dataset, Kaluva et al. (22) reached global DSC of 0.92 and 0.62 on liver and tumor, respectively. Pandey et al. (30) obtained a DSC of 0.59 on liver tumor segmentation by introducing ResNet-blocks and reducing the deep neural network’s complexity. Compared to these results our proposed approach outperforms them. When liver tumors are < 1cm in diameter, CT is a less sensitive modality than hepatobiliary MRI with specific contrast enhancers (such as gadoxetate), which is preferred in clinical practice (31). For the detection of such small liver lesions, CT and MRI should be used in combination for guidance in pre-ablation treatment. Therefore, in our study we performed a sub-analysis to validate the performance of the model on tumors > 1cm in diameter which correspond to an approximate volume of 0.5 cm^3^. The model demonstrated a higher detection and segmentation performance for tumors with a volume ≥ 0.5 cm^3,^ which meets the clinical practical application requirements very well. The performance improvement was consistent with prior studies (32–34).

In our study, the input size for liver segmentation was 512×512×z, where z was the number of slices per CT subject, and the input size for a tumor or ablation segmentation was 20×30×30 or 40×60×60. For liver segmentation, using 3D RA-Unet with larger 3D patches would be computationally much more expensive than 2D RA-Unet due to its complexity. Besides, the 2D liver segmentation model had given us an acceptable result (the dice of liver segmentation was close to 95%), so we choose 2D RA-Unet for liver segmentation. For tumor segmentation, to retain sufficient spatial information and improve the segmentation accuracy of these small lesions, we used 3D RA-Unet to segment the tumor and the ablation area.

Accurate segmentation of the ablation zone helps assess the minimal ablative margin, which has a significant influence on the prognosis of patients with malignant liver tumors, and may determine the treatment plan to follow (35, 36). To the best of our knowledge, we have been the first to automatically segment the ablative zone in patients after RFA/MWA treatment. In our investigation, the ablation zone model achieved a high segmentation performance with a median DSC of 0.83 on the arterial phase and 0.89 on the portal venous phase. The ability to precisely assess the ablation zone is necessary to verify complete tumor ablation including a sufficient safety margin. The method presented in this paper may be the basal step for a fully automated MAM assessment pipeline in clinical practice.

Many studies have developed liver and liver tumor segmentation algorithms only on the portal venous phase (19, 22, 30). However, a generalized algorithm that is robust across multiple phases would be beneficial and practical for clinical applications. There are various enhancing patterns of hepatic lesions according to their blood supply characteristics (37). Some tumors tend to be more evident in the arterial phase in the liver context, e.g., HCC, and others, the majority of metastases, tend to be evident in the portal venous phase. It is critical to select the right phase to visualize the most apparent tumor boundary for further evaluation. In real clinical practice, doctors can interactively select appropriate phases to automatically segment lesions of different characteristics, which yield greater efficiency. Our study shows that transfer learning can be an effective approach for training CNNs on data from routine clinical practice.

The initial CNN was trained using 130 CT scans from LiTS dataset, which included only portal venous phase images. We showed that using transfer learning, an initial model can be generalized to another imaging phase with a relatively small amount of additional training data. We used both the arterial and portal venous phase images from the local clinical dataset to train the model. The transfer learned models demonstrate good performances on the arterial phase images with a median DSC of 0.95 for the pre-ablation group, and 0.96 for the post-ablation group for liver segmentation, 0.64 for liver tumor segmentation, and 0.83 for ablation zone segmentation. This indicates that the amount of training data for a specific task does not need to increase linearly with the number of images. For CNN training, the two-step phased approach may be practical, especially when clinical data is limited. The liver tumor segmentation model showed weaker performance in the arterial phase than in the venous phase, suggesting that different phase images had a more significant impact on tumor segmentation tasks than liver and ablation zone segmentation. We speculate that the hepatic enhancement in the arterial phase is not sufficient, making the tumor’s edge slightly fuzzy, which increases the difficulty for tumor segmentation. More research and data may be required to further improve the accuracy of the liver tumor segmentation model on the arterial phase.

## Limitations

Our study has a few limitations. Firstly, it relies on retrospective data from a single-center. Secondly, the liver tumor and ablation zone segmentations need to be assessed from a clinical treatment perspective. The performance and value of the proposed methods in a fully automated MAM assessment pipeline need to be further evaluated. Thirdly, the small number of study patients in the clinical dataset is a potential drawback of this study. Nevertheless, the results are encouraging and justify the approach. In the future, evaluation of the proposed deep learning method using multi-center data with different scanners and protocols should be conducted.

## Conclusion

The proposed deep learning approach can provide automated segmentation of liver tumors and ablation zones on multi-phase (arterial and portal venous) and multi-time-point (before and after RFA/MWA ablation) routine clinical CT images, enabling quantitative evaluation of treatment success. Using transfer learning, an initial model can be generalized to another imaging phase and another type of lesion with a relatively small amount of additional training data.

## Data Availability Statement

The original contributions presented in the study are included in the article/supplementary material. Further inquiries can be directed to the corresponding author.

## Ethics Statement

The studies involving human participants were reviewed and approved by Faculty of Medicine and University Hospital Cologne. The patients/participants provided their written informed consent to participate in this study.

## Author Contributions

KH, XL, and RS contributed to conception and design. KH and RR organized the data and performed annotations. RR, CW, and JN acquired the data. KH wrote the first draft of the manuscript. XL, RS, and FT developed the deep learning model and performed the statistical analysis. AB, HZ, and MP supervision, study design. All authors contributed to the article and approved the submitted version.

## Funding

This study has received funding by Jilin Provincial Key Laboratory of Medical imaging & big data (20200601003JC), Radiology and Technology Innovation Center of Jilin Province (20190902016TC), Jilin Province Science and Technology Department Science and Technology Innovation Talents Cultivation Program (20180519008JH), National Natural Science Foundation of China and German Research Foundation (C-0007), China International Medical Foundation (Z-2014-07-2003-03), and Northeast university-Key Laboratory of Intelligent Computing in Medical Image, Ministry of Education (3D4206967428).

## Conflict of Interest

The authors declare that the research was conducted in the absence of any commercial or financial relationships that could be construed as a potential conflict of interest.
